# Effect of Addition of Ca^2+^ and CO_3_^2−^ Ions with Temperature Control on Self-Healing of Hardened Cement Paste

**DOI:** 10.3390/ma12152456

**Published:** 2019-08-01

**Authors:** Heesup Choi, Masumi Inoue, Dongmin Kim, Hyeonggil Choi, Risa Sengoku

**Affiliations:** 1 Department of Civil and Environmental Engineering, Kitami Institute of Technology, Hokkaido 090-8507, Japan; 2 Department of Infrastructure Safety Research, Korea Institute of Civil Engineering & Building Technology, Gyeonggido 10223, Korea; 3 School of Architecture, Kyungpook National University, Daegu 41566, Korea; 4 Research & Development Center, Taiheiyo Cement Corporation, Chiba 285-8655, Japan

**Keywords:** cementitious materials, self-healing, CaCO_3_, nanosized ultrafine CO_2_ bubble, Ca^2+^, CO_3_^2−^, temperature, pH, vaterite

## Abstract

Concrete has a remarkably low ratio of tensile strength to compressive strength, and is widely used in construction. However, the occurrence of cracks in a concrete structure is inevitable. Nevertheless, in the presence of adequate moisture, small cracks in the concrete structure exhibit a propensity to self-heal by getting filled due to the rehydration of cement particles and the subsequent precipitation of calcium carbonate (CaCO_3_). According to previous studies, the self-healing performance can be maximized by optimizing the temperature and pH to control the crystal formation of CaCO_3_. This study focused on the crystal form of CaCO_3_ generated in the self-healing of a cement-based composite material. To evaluate the self-healing performance depending on the type of aqueous solution and the temperature, the weight change, the weight change rate, and the porosity reduction in each case were evaluated. Moreover, to increase the generation of CaCO_3_ (which is a self-healing precipitate), nanosized ultrafine CO_2_ bubbles using CO_2_ gas were used, along with an adequate supply of Ca^2+^ by adjusting the aqueous solution (Ca(OH)_2_, CaO + ethanol). For greater pore-filling effects by controlling the CaCO_3_ crystal forms in the cement matrix, the change in the crystal form of the precipitated CaCO_3_ in the hardened cement paste with changing temperature was analyzed by scanning electron microscopy and X-ray diffraction. As a result, the possibility of the effective generation and control of vaterite with a dense pore structure together with calcite was confirmed by adjusting the temperature to approximately 40 °C at a pH of 12.

## 1. Introduction

**Figure 1:**
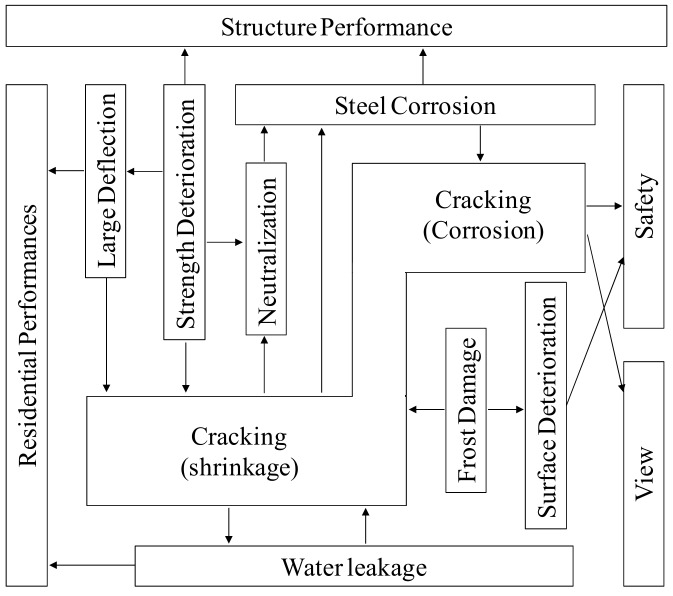
Correlation of degradation with cracking.

Concrete is the most widely used material in the field of construction and has no viable replacement in the foreseeable future. However, since its tensile strength is remarkably low when compared to its compressive strength, the occurrence of large and small cracks in a concrete structure is inevitable [1]. The cracks generated in such a concrete structure greatly increase ion diffusion inside the concrete, as well as the water permeability. Moreover, the cracks serve as infiltration points for external elements such as water, oxygen, chloride, sulfate, and carbon dioxide, which catalyzes the process of deterioration [2,3,4]. Additionally, they compromise the safety, usability, durability, and appearance of a structure, as shown in Figure 1. Hence, it is important to prevent the formation of detrimental fine cracks in order to preserve concrete structures for longer periods of time. In Japan, cracks in concrete, which do not exceed the allowable crack width, are not prioritized for rectification as far as structural durability is concerned [5]. Although these fine cracks do not pose an immediate problem, they deteriorate progressively and compromise the safety and integrity of the concrete structure, and may lead to their eventual failure if left unchecked [6,7,8]. Accordingly, for a concrete structure built using a cement-based water-tight composite material, it is imperative to prevent the occurrence of fine cracks at the initial stage itself.

In an aqueous environment, it has been observed that relatively finer cracks in the concrete undergo self-healing, where a part of the crack is filled due to the rehydration of cement particles and the subsequent precipitation of CaCO_3_ [9]. Self-healing products usually contain hydrates such as C–S–H, ettringite, and calcium hydroxide (Ca(OH)_2_), which precipitate along with CaCO_3_ at the newly generated crack surfaces [10,11]. In the self-healing mechanism of concrete, CaCO_3_ is produced as a carbonic acid that is not readily dissolved in water due to the reaction of the Ca^2+^ in concrete with CO_3_^2−^ dissolved in water [12,13,14]. This is the chemical process by which cracks undergo self-healing (i.e., cracks whose widths are less than 0.1 mm) [12,15,16]. The deposition of CaCO_3_ is reported to occur according to the following reactions Equations (1)–(3) [12].

H_2_O + CO_2_⇔H_2_CO_3_⇔H^+^ + HCO_3_^−^⇔2H^+^ + CO_3_^2−^ (1)

Ca^2+^ + CO_3_^2−^⇔CaCO_3_    (pH_water_ > 8) (2)

Ca^2+^ + HCO_3_^−^⇔CaCO_3_ + H^+^  (7.5 < pH_water_ < 8) (3)

Among the many studies related to self-healing [17,18,19,20,21,22,23,24,25], Choi et al. (2017) reported that carbon dioxide was produced as ultrafine nanosized CO_2_ bubbles due to self-healing under aqueous conditions [26]. The CO_2_ precipitated large amounts of CaCO_3_ in the surface layer of the concrete, and inside the micro-cracks due to the self-healing of cement-based composite materials. Most precipitates were identified as vaterite [26,27,28]. Representative crystals of CaCO_3_ can be classified into three types, including calcite, vaterite, and aragonite. Almost all the CaCO_3_ produced as Ca(OH)_2_ in the hardened specimen of cement combined with CO_3_^2−^ in the pore water can be classified as calcite [26,29,30]. In particular, the density of vaterite is slightly lower than that of calcite. Nevertheless, the crystal size of vaterite as a stable hexagon of the crystal structure is smaller than that of calcite. Therefore, vaterite exhibits excellent pore-filling effects superior to those of other CaCO_3_ crystal forms [26,29,30,31]. According to previous studies, crystal polymorphism of CaCO_3_ can be controlled to produce vaterite with denser pore-filling effects than calcite at a pH of 9.0 or at a water temperature of 30 °C to 50 °C [26,28,29,30].

**Figure 2:**
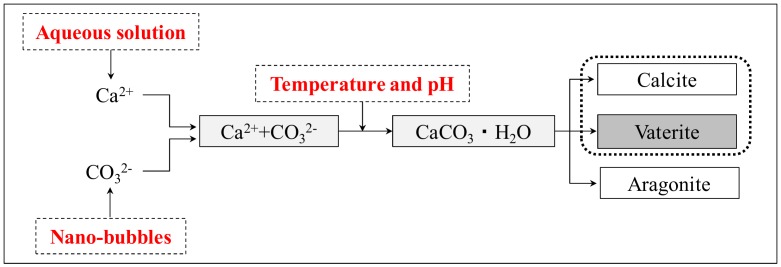
Process of self-healing by temperature control.

The objective of this study is to focus on the crystal forms of CaCO_3_ generated in the self-healing of hardened cement paste and determine the effect of temperature on the crystal forms of CaCO_3_, which is precipitated during self-healing. For greater pore-filling effects by controlling CaCO_3_ crystal forms in the cement matrix, an optimum temperature of the aqueous solution is required to enable the generation of a large quantity of vaterite together with calcite, among the other products obtained by self-healing. Moreover, to increase the production of CaCO_3_ as a precipitate, a study was conducted using nanosized ultrafine CO_2_ bubbles (from CO_2_ gas), in addition to an adequate supply of Ca^2+^ ions, by optimizing each aqueous solution. The self-healing process of this study is shown in Figure 2.

## 2. Materials and Methods

### 2.1. Materials and Specimen Overview

**Figure 3:**
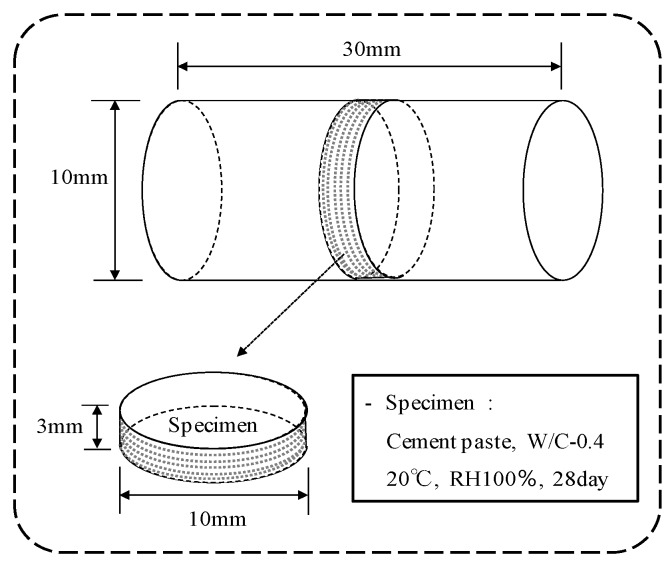
Schematic of the preparation of the cement samples.

The major reaction materials generated during the self-healing of cement-based composite materials are controlled by the hydration reaction between cement particles and water [12]. Thus, the author evaluated self-healing performance using cement paste in this experiment. Using ordinary Portland cement (C, density: 3.16 g/cm^3^, Average particle diameter: 10 μm) according to ASTM C 150 [32], specimens were prepared with a water-cement ratio of 0.4. The particle size distribution of the cement was measured using a laser diffraction particle size analyzer (SALD-30000, Shimadzu Ltd., Tokyo, Japan). The specimen of 10 × 30 mm^2^ was sealed after it was produced and after it was cured at a constant temperature and humidity of 20 ± 1 °C and 80% in a thermostatic chamber for 24 h. It was cured in a tank of 20 ± 1 °C water from 2 days to 28 days of material age. After then, the specimens were cut into φ 10 × 3 mm pieces using a cutter and used prior to self-healing, as shown in Figure 3.

### 2.2. Experimental Methods

**Figure 4:**
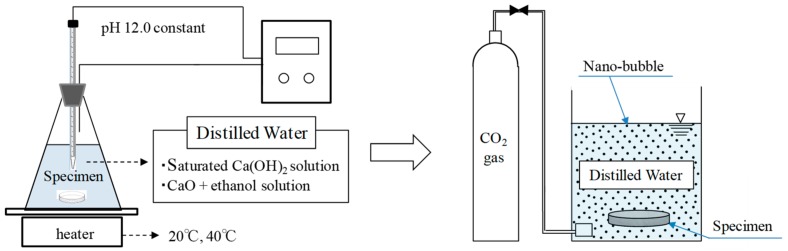
Schematic of experimental protocol for self-healing.

Table 1 shows the experimental factors and conditions of this experiment and Figure 4 shows the schematic diagram of the experimental protocol. For self-healing, an aqueous solution of saturated calcium hydroxide (CH, Ca(OH)_2_ solution) and an aqueous solution prepared by mixing calcium oxide (CaO) and ethanol (C_2_H_5_OH) (CE, CaO + ethanol solution) were used as an additional source of Ca^2+^. According to previous studies [33], the aqueous solution prepared by mixing calcium oxide and ethanol promotes the reaction between Ca^2+^ and CO_3_^2−^ by delaying the reaction between Ca^2+^ and OH^−^, which enhances the generation of calcium carbonate. In this case, the CE aqueous solution was prepared by mixing ethanol with calcium oxide to obtain 0.5 mol-ethanol/mol-CaO and agitating it with distilled water [33]. Moreover, to increase the supply of CO_3_^2−^, nanosized (average particle diameter: 50 nm) ultrafine CO_2_ bubbles were supplied using an ultrafine bubble generating device that operates on the principle of cavitation [26,28]. The nanosized ultrafine CO_2_ bubbles were measured using a zeta potential and particle size analyzer (ELSZ-2000, Otsuka Electronics Co., Ltd., Tokyo, Japan). The experiment was carried out at two temperatures while maintaining the pH of the solution at 12. The first experiment was performed at 20 °C (at which calcite was mainly generated), while the second one was performed at 40 °C (at which vaterite was mainly generated). These temperatures were chosen according to the existing literature reports of the temperatures at which each crystal form of CaCO_3_ is generated [29,30].

The specimens were cut into sizes of φ 10 × 3 mm, and were first immersed in aqueous solutions maintained at 20 °C and 40 °C, respectively, for a prescribed time, as shown in Figure 4. Subsequently, all specimens were immersed in an aqueous solution (distilled water) for 4 h, during which nanosized CO_2_ bubbles were generated. In this case, the experiment was carried out under four conditions: 5, 10, 15 and 20 h of immersion times in CH and CE aqueous solutions each, irrespective of the temperature (Table 1). Subsequently, the specimen prior to self-healing with curing in water at 20 °C for 28 days was compared with the specimen after self-healing to analyze the self-healing performance.

### 2.3. Characterization

Table 2 shows the sequence and method of experiment. To evaluate the change in the physical properties of the hardened cement paste resulting from self-healing and the self-healing precipitate, a comparative evaluation was carried out on each condition prior to self-healing (A) and after self-healing (B).

First, to evaluate the self-healing performance depending on the type of aqueous solution, the weight change and weight change rate were calculated from the absolute dry weights of the specimens prior to and after self-healing in all the cases depending on the difference in immersion times. Note that hardened cement paste is a porous structure. Therefore, to evaluate the change in the number of pores in the specimen prior to and after self-healing, the porosity reduction resulting from the changes in the weight and the absorption rate prior to and after self-healing were calculated using the IV specimen (24 h). In the meantime, the pore reduction rate was calculated using the correlation between the weight change of the specimen prior to and after self-healing and the theoretical model [34] of the amount of hydration product and porosity, as proposed by Papadakis. The filling effect of self-healing resulting from the change in the crystal form of CaCO_3_ was quantitatively evaluated by comparing it with the porosity reduction resulting from the actual weight change.

The crystals of CaCO_3_ are the main precipitates of self-healing. Among them, to obtain vaterite, which has better pore-filling effects and a more stable crystal structure than calcite [29,30], scanning electron microscopy (SEM) and X-ray diffraction (XRD) analyses were performed for each case at different temperatures.

**Figure 5:**
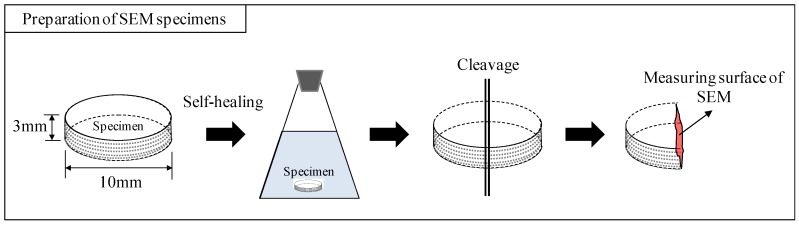
Sample preparation for SEM.

SEM analysis was performed on the cleaved surface of the specimens (each surface sized φ 10 × 3 mm) (Figure 5). The crystal forms and sizes of the experimentally obtained CaCO_3_ were evaluated and compared with the data from existing literature [29,30]. For the SEM observations, the JSM-6380, manufactured by JEOL Ltd. (Tokyo, Japan), was used to examine the samples at 5000x, 10,000x and 20,000x magnifications at a voltage of 15 kV. The samples used in the SEM observations were coated with platinum.

XRD analysis was performed using the Rigaku Smart LabX (Tokyo, Japan) under the following conditions: a tube voltage of 40 kV, tube current of 20 mA, scanning range of 2*θ* = 5° to 65°, scan step of 0.02°, and scan speed of 1°/min. In addition, 5 wt % alpha-alumina (α-Al_2_O_3_) was used as the internal standard substance.

Furthermore, hydration was stopped on some specimens by immersing them in acetone after the completion of self-healing. These specimens were left stationary for 7 days in an environment of 11% relative humidity, and were, subsequently, analyzed by scanning electron microscopy (SEM) and X-ray diffraction (XRD).

## 3. Results and Discussion

### 3.1. Absolute Dry Weight Ratio Change by Self-Healing

To identify changes in the physical characteristics of the hardened cement paste according to the type of aqueous solution and temperature, the differences in the absolute dry, surface-dried, and underwater weights prior to and after self-healing were used to calculate the absolute dry weight ratio (%) and the porosity reduction by self-healing (%).

Table 3 shows the increased absolute dry weights of the CH (Ca(OH)_2_) and CE (CaO + ethanol) series when each specimen (three samples each) underwent self-healing. Referring to Table 3, and using the results for the first sample of the specimen, the absolute dry weight ratio and porosity reduction by self-healing of the CH and CE series were evaluated and compared.

**Figure 6:**
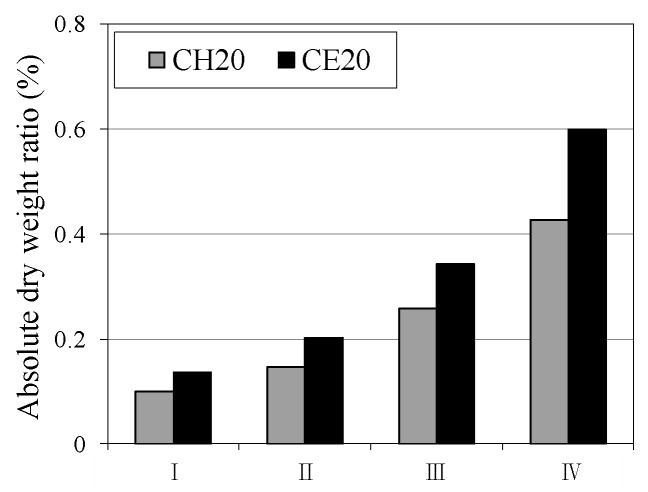
Absolute dry weight ratio (20 °C).

**Figure 7:**
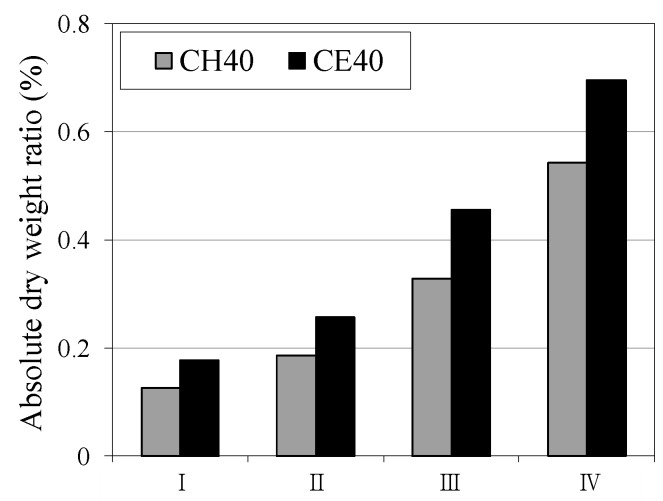
Absolute dry weight ratio (40 °C).

Figure 6 and Figure 7 show the changes in the weight ratio depending on the type of aqueous solution and self-healing period. In this case, the weight ratio change in each case was calculated by subtracting the weight of the specimen prior to self-healing from the weight of the specimen after self-healing. It was observed that, as the self-healing period increases, the specimens of each aqueous solution showed a common trend of weight ratio increase irrespective of the aqueous solution temperature, compared to prior self-healing. In addition, the weight ratio of the specimen increased by 1.2 times when the temperature of the aqueous solution was increased from 20 °C to 40 °C. In particular, as shown in Figure 7 when the temperature of the aqueous solution was 40 °C, the specimen immersed in the CE (CaO + ethanol) aqueous solution showed a weight ratio increase of ~1.3 times as compared to the weight ratio immersed in the (Ca(OH)_2_) aqueous solution.

**Figure 8:**
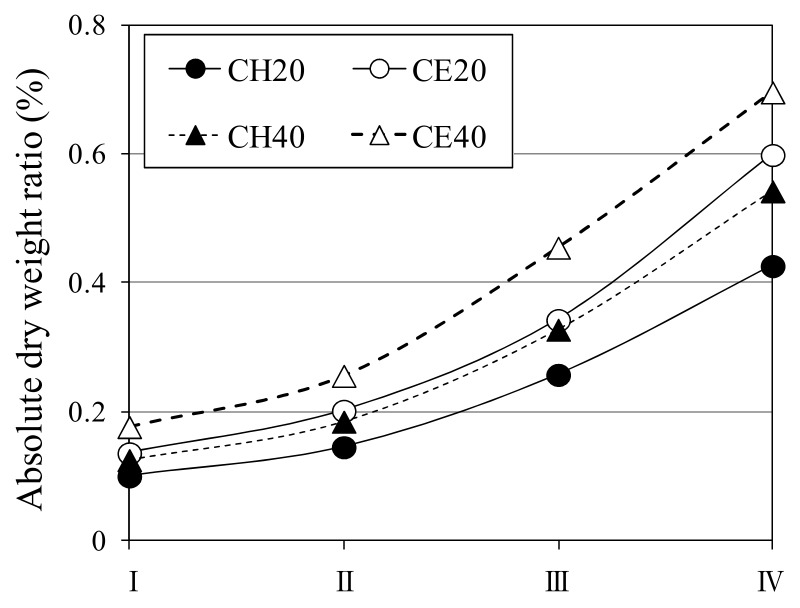
Absolute dry weight ratio for different solutions and temperatures.

Figure 8 shows the rate at which the weight ratio increases after self-healing under each condition. In general, as the self-healing period increased, the rate of the weight ratio increase showed an increasing trend. In particular, for a self-healing period IV (24 h), the rate of weight ratio increase obeyed the following trend: CE40 > CE20 > CH40 > CH20.

Hence, it can be inferred that increasing the temperature of the aqueous solution increases the rate of the reaction between Ca^2+^ and CO_3_^2−^, even if the amount of Ca^2+^ supplied to each aqueous solution is fixed. Thus, the rate of the reaction between Ca^2+^ and CO_3_^2−^ (and in turn, precipitation of CaCO_3_) is higher at 40 °C than at 20 °C. Additionally, the increase in weight was shown to be much higher in the CE series than that in the CH series, since the reaction between Ca^2+^ and CO_3_^2−^ was promoted by using calcium oxide and ethanol as the self-healing solution, which further improves the self-healing performance.

### 3.2. Change in Porosity Reduction by Pore Filling of Self-Healing

To review the CaCO_3_ filling effect using the dry weights of CH and CE series on the basis of the case of self-healing period IV (24 h) prior to and after self-healing, the porosity reduction $∆P\left[\%\right]$ after self-healing was obtained by using Equation (4).

(4) $$∆P=\frac{P_{A}-P_{B}}{P_{A}}\times 100$$

In this case, $P_{A}$ (%) is the porosity before self-healing (A) and $P_{B}$(%) is the porosity after self-healing (B).

To microscopically review the CaCO_3_ filling effect using the actual weight change, the porosity reduction after self-healing, due to the generation of CaCO_3_, was obtained by using the theoretical model proposed by Papadakis, wherein the amount of hydration product generated and its porosity are considered [34].

In this case, if Ca(OH)_2_ (molar mass = 74 [g/mol]) is converted to CaCO_3_ (molar mass = 100 [g/mol]), the weight increases by 26 g per mol [35]. Based on the previously mentioned theoretical model [34], the total weight increase $W_{total}\;$[g] and the reaction rate R[%] in the case where Ca(OH)_2_ is entirely converted to CaCO_3_ after self-healing of the CH and CE series for each temperature can be obtained using Equations (5) and (6).

(5) $$W_{total}=\left(100-74\right)\cdot \left[Ca{\left(OH\right)}_{2}\right]\cdot \;\left(W_{d}+∆W_{d}\right)$$

(6) $$R=\frac{∆W_{d}}{W_{total}}\times 100$$

In this case, $W_{d}$ is the weight [g] before self-healing (A) and $∆W_{d}$ is the weight increase or decrease [g] after self-healing (B).

Moreover, the concentration of Ca(OH)_2_ was calculated on the basis of Equations (7) and (8) proposed by Papadakis [36,37].

(7) $$\left[Ca{\left(OH\right)}_{2}\right]=\frac{3}{2}{\left[C_{3}S\right]}_{0}F_{C_{3}S}+\frac{1}{2}{\left[C_{2}S\right]}_{0}F_{C_{2}S}-4{\left[C_{4}AF\right]}_{o}F_{C_{4}AF}-{\left[C_{3}A\right]}_{0}F_{C_{3}A}+{\left[C\bar{S}H_{2}\right]}_{0}$$

(8) $$F_{i}\left(t\right)=1-\frac{\left[i\right]}{{\left[i\right]}_{0}}=1-{\left(1-k_{H,i}\;t\left(1-n_{i}\right)\right)}^{1/\left(1-n_{i}\right)}$$

In this case, $F_{i}\left(t\right)$ is the reaction rate of substance *i* at time *t*, $\left[i\right]$ and ${\left[i\right]}_{0}$ are the concentration of substance *i* at time *t* and the initial concentration of substance *i* [mol/m^3^], respectively. Additionally, $k_{H,i}$ is the reaction rate constant [1/s] of substance *i* at 20 °C, and $n_{i}$ is the value obtained in the experiment.

Lastly, to obtain porosity reduction $∆\in_{C_{B}}$ resulting from the generation of CaCO_3_ after self-healing using the actual weight change, the author used Equation (9) as proposed by Papadakis, to which the porosity reduction by carbonation is applied [34].

(9) $$∆\in_{C_{B}}=\frac{∆\in_{C}\cdot \left(\frac{R}{100}\right)}{\in_{0}-∆\in_{H}\left(t\right)}\times 100$$

In this case, $\in_{0}$ (%) is the initial porosity of fresh concrete, $∆\in_{H}\left(t\right)$ (%) is the porosity by a hydration reaction, and $∆\in_{C}$ (%) is the porosity reduction resulting from the generation of CaCO_3_.

**Figure 9:**
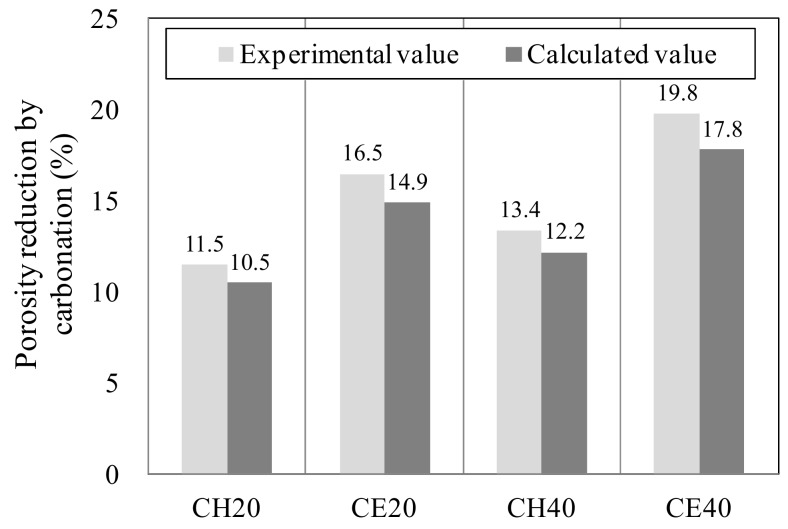
Porosity reduction by carbonation (IV series).

Figure 9 shows the porosity reduction resulting from the generation of CaCO_3_ following the temperature change after self-healing of the CH and CE series on the basis of the case of the self-healing period IV (24 h). In this scenario, the experimental value (∆P) of porosity was used, and the calculated value ($∆\in_{C_{B}}$) was obtained through the weight change and the theoretical model of Papadakis [34].

According to the experimental results, although the experimental porosity values (∆P) of CH 20, CE 20, CH 40, and CE 40 after self-healing (Step B) decreased by about 1.0%, 1.6%, 1.2%, and 2.0%, respectively, when compared to the calculated values ($∆\in_{C_{B}}$) irrespective of the temperature change, the differences were not significant. Meanwhile, when the temperature of the aqueous solution was 20 °C, the experimental and theoretical values of the CE series increased by about 5.0% and 4.5%, respectively, when compared to those of the CH series. Additionally, when the temperature of the aqueous solution was 40 °C, the experimental and theoretical values of the CE series increased by about 6.5% and 5.5%, respectively, when compared to those of the CH series.

Therefore, it may be inferred that, when the temperature of the aqueous solution was maintained at about 40 °C, the amount of CaCO_3_ generated in the CE series (which uses the aqueous solution prepared by mixing ethanol with calcium oxide) increased as compared to that generated in the CH series (which uses the aqueous solution of calcium hydroxide), due to the elevated rate of reaction between Ca^2+^ and CO_3_^2−^ at a high temperature. From the difference in the number of pores before and after self-healing (experimental value), it may be possible to theoretically predict the change in the number of pores resulting from the generation of CaCO_3_ after self-healing, by using the weight change and Papadakis’ model. However, when this model was applied to this study, the evaluation was carried out assuming that only Ca(OH)_2_ contributes to a porosity change resulting from carbonation in the specimens subjected to self-healing. Thus, when the number of pores are compared in the specimens before and after self-healing, the difference of about 1% to 2% was observed on the basis of the actual absorption rate of this experiment. Therefore, the change in the number of pores resulting from the carbonation of the hydration product except Ca(OH)_2_, such as C–S–H gel, ettringite, and Friedel’s salt, after self-healing, must be quantitatively reviewed.

### 3.3. Crystallographic Change in Calcium Carbonate (CaCO_3_) by Scanning Electron Microscopy (SEM) and X-Ray Diffraction (XRD)

The aim of this experiment was to confirm the possibility of controlled vaterite (one of the polymorphs of CaCO_3_) generation by optimizing the temperature of the aqueous solution during self-healing. For this purpose, Scanning Electron Microscopy (SEM) and X-ray Diffraction (XRD) analyses were performed at each temperature (20 °C and 40 °C) using the CE series on the basis of the case of the self-healing period IV (24 h).

**Figure 10:**
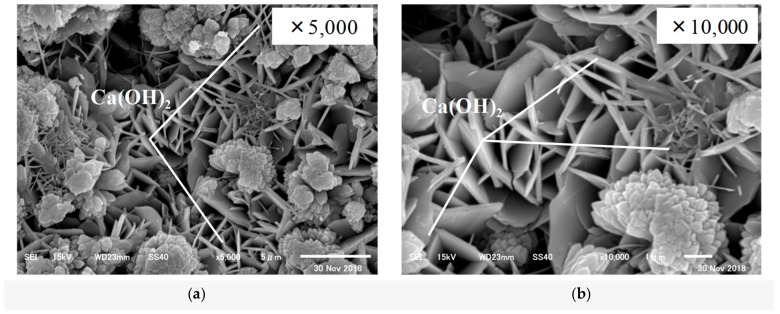
Hydration products before self-healing (**a**) 5000x; (**b**) 10,000x.

**Figure 11:**
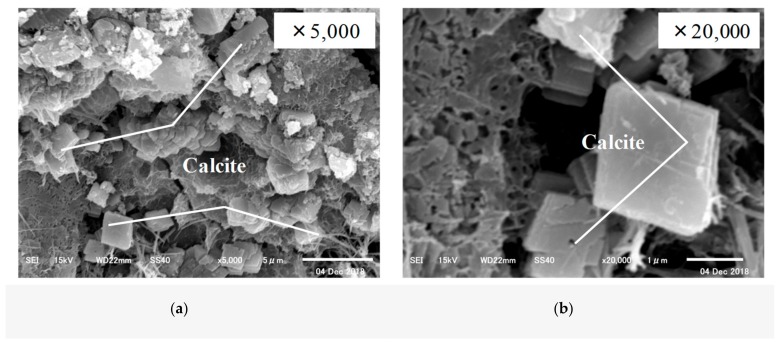
Self-healed substances at 20 °C (CE specimen of IV series) (**a**) 5000x; (**b**) 20,000x.

**Figure 12:**
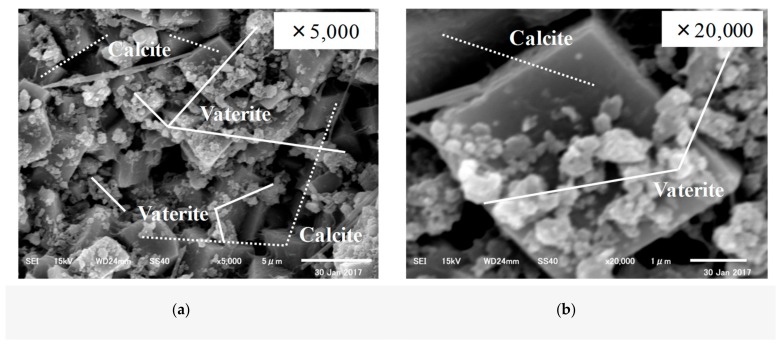
Self-healed substances at 40 °C (CE specimen of IV series) (**a**) 5000x; (**b**) 20,000x.

First, SEM analysis was carried out on the CE solution. The SEM images of the specimens before and after self-healing at 20 °C and 40 °C are shown in Figure 10, Figure 11 and Figure 12. 

The SEM analysis results showed that the specimen before self-healing in Figure 10 showed the presence of mostly Ca(OH)_2_ and C–S–H, while there was almost no CaCO_3_ present among the cement hydrates. In the case of the specimen after self-healing in Figure 11, for which the temperature of the aqueous solution was adjusted to 20 °C, calcite (the most stable polymorph of CaCO_3_) was observed along with C–S–H. In addition, in the case of the specimen after self-healing in Figure 12, for which the temperature of the aqueous solution was adjusted to 40 °C, vaterite (which is the target of this study) was mainly generated and found to be mainly attached to the surface of the calcite.

**Figure 13:**
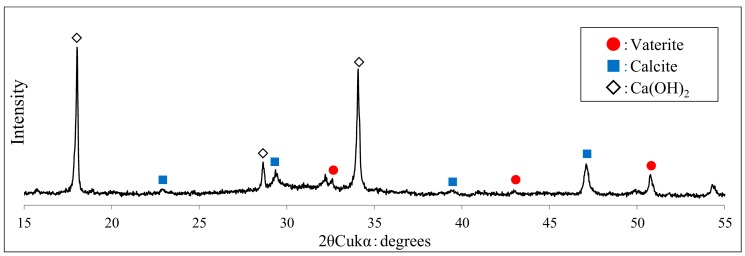
XRD patterns at 20 °C (CE specimen of IV series).

**Figure 14:**
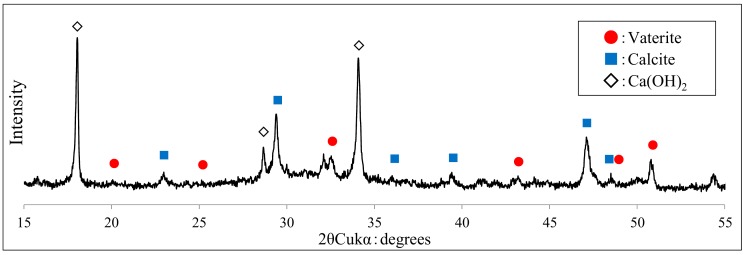
XRD patterns at 40 °C (CE specimen of IV series).

After SEM analysis of the generated CaCO_3_ crystals from the specimens after self-healing, XRD analysis was carried out using the specimens identical to those of SEM. The XRD analysis results of the specimens after self-healing in the aqueous solution at 20 and 40 °C are shown in Figure 13 and Figure 14, respectively. In addition, for XRD analysis of this study, by referring to the peaks of crystal polymorphism of Ca(OH)_2_ and CaCO_3_ (calcite and vaterite) previously reported [38,39], identification of hydration products and self-healing precipitates was carried out to range from 15° to 55°.

As shown in Figure 13 and Figure 14, XRD analysis confirmed that the crystal forms of CaCO_3_, identified n the SEM analysis, were mostly calcite in the case of 20 °C and mostly vaterite along with some calcite in 40 °C. Additionally, when the self-healing aqueous solution temperature was 40 °C, vaterite peaks were more numerous as compared to those in the case of 20 °C. Additionally, the peak intensity of each crystal was found to be higher at 40 °C. Hence, it may be inferred that the generation of vaterite increased along with that of calcite as the temperature increased from 20 to 40 °C.

Accordingly, if self-healing is carried out by controlling the temperature of the aqueous solution, the crystal forms of CaCO_3_ generated in the cement matrix are mostly calcite in the case of 20 °C. In the case of 40 °C, they can be controlled so that the major part of the obtained crystal forms are vaterite along with some calcite. In particular, at a pH of 12, vaterite with significant pore-filling effects can be effectively generated and controlled by adjusting the temperature of the aqueous solution at about 40 °C. Additionally, the pore structure can be made denser, and the self-healing performance can be improved by attaching vaterite to the surface of calcite, whose crystal size is larger than that of vaterite.

## 4. Conclusions

In this study, the focus was on the crystal forms of CaCO_3_ generated during self-healing and the possibility of modifying them in the hardened cement paste by adjusting the temperature of the aqueous solution to obtain vaterite, which shows significant pore-filling effects and has a more stable crystal structure than that of calcite. The temperature that enables the generation of a large amount of vaterite along with calcite among the crystals of CaCO_3_ was also determined. The results of the study are summarized below.

(1) By fixing the amount of Ca^2+^ supplied to each aqueous solution, it has been confirmed that the higher the temperature, the higher is the rate of reaction between Ca^2+^ and CO_3_^2−^. Thus, the rate of reaction is more for the aqueous solution maintained at 40 °C, as compared to that at 20 °C. Thus, the generation of CaCO_3_ after self-healing increased.

(2) In addition, when self-healing is carried out using the aqueous solution of CE, the rate of the reaction between Ca^2+^ and CO_3_^2−^ is higher as compared to that for the aqueous solution of CH, which implies that the precipitation of CaCO_3_ is enhanced after self-healing, irrespective of the temperature. Thus, the absolute dry weight ratio and porosity reduction may increase, and more effective healing is possible.

(3) Considering that the difference between the porosity reduction based on the actual absorption rate and the weight change is not significant, the change in the number of pores by self-healing can be evaluated and the filling effect of self-healing can be predicted by applying the actual weight change before and after self-healing, according to the theoretical model proposed by Papadakis.

(4) In the case of self-healing using the aqueous solution of CE, by controlling the temperature, the crystal forms of CaCO_3_ generated in the cement matrix can be changed mostly to calcite when the solution is maintained at 20 °C and to a large amount of vaterite along with calcite at 40 °C. At a pH of 12, vaterite (which shows significant pore-filling effects and more stable crystal structure than calcite) can be effectively generated by adjusting the temperature of the aqueous solution to about 40 °C.

(5) Additionally, the pore structure can be made denser and the self-healing performance can be improved by attaching vaterite on the surface of calcite, whose crystal size is larger than that of vaterite.

## Figures and Tables

**Table 1:** Experimental factors and conditions.

Specimen: Hardened Cement Paste (Water/Cement Ratio: 0.4)			
Self-Healing Condition [29,30]	Temperature: 20 °C and 40 °C (Constant pH of 12)	Ca(OH)_2_ + CO_2_ Nano-bubble (CH) CaO + Ethanol + CO_2_ Nano-bubble (CE)	
Self-healing period	Prior to self-healing		
	After self-healing	CH, CE (5 h) + CO_2_ nano-bubble (4 h)	Ⅰ
		CH, CE (10 h) + CO_2_ nano-bubble (4 h)	Ⅱ
		CH, CE (15 h) + CO_2_ nano-bubble (4 h)	Ⅲ
		CH, CE (20 h) + CO_2_ nano-bubble (4 h)	Ⅳ

Note: CH: Ca(OH)_2_ solution. CE: CaO + ethanol solution.

**Table 2:** Experimental procedure and evaluation.

Step	Experimental Sequence	Subject and Method of Evaluation	
		Physical Property Change	Self-Healing Substances
A	Prior to self-healing	-Absolute dry weight -Absolute dry weight ratio -Porosity reduction by carbonation	-SEM -XRD
B	After self-healing		

Note: A: Prior to self-healing. B: After self-healing (I, II, III, IV).

**Table 3:** Increased absolute dry weight of the CH and CE series by self-healing.

Temp.	Type	Increased Absolute Dry Weight (g)											
		1st				2nd				3rd			
		Ⅰ	Ⅱ	Ⅲ	Ⅳ	Ⅰ	Ⅱ	Ⅲ	Ⅳ	Ⅰ	Ⅱ	Ⅲ	Ⅳ
20 °C	CH20	0.018	0.026	0.046	0.076	0.016	0.023	0.044	0.076	0.018	0.024	0.047	0.077
	CE20	0.025	0.060	0.078	0.090	0.021	0.058	0.074	0.088	0.024	0.059	0.080	0.089
40 °C	CH40	0.024	0.046	0.078	0.088	0.025	0.045	0.080	0.087	0.026	0.047	0.077	0.091
	CE40	0.035	0.072	0.096	0.123	0.033	0.070	0.095	0.119	0.033	0.069	0.101	0.124

Note: CH20: Temperature of Ca(OH)_2_ solution = 20 °C. CE20: Temperature of CaO+ethanol solution = 20 °C. CH40: Temperature of Ca(OH)_2_ solution = 40 °C. CE40: Temperature of CaO + ethanol solution = 40 °C. 1st, 2nd, and 3rd: number of repetition measurements. I, II, III, and IV: self-healing period. I: CH, CE (5 h) + CO_2_ nano-bubble (4 h). II: CH, CE (10 h) + CO_2_ nano-bubble (4 h). III: CH, CE (15 h) + CO_2_ nano-bubble (4 h). IV: CH, CE (20 h) + CO_2_ nano-bubble (4 h).
